# Supplementary material for: Evaluating the effectiveness of ensemble voting in improving the accuracy of consensus signals produced by various DTWA algorithms from step-current signals generated during nanopore sequencing
Source: PLoS Comput Biol. 2021 Sep 10;17(9):e1009350. doi: 10.1371/journal.pcbi.1009350 (PMC8457506; doi:10.1371/journal.pcbi.1009350)
Supplement: Attachment — Submitted filename: Response to reviewers comments_27July2021.docx [file pcbi.1009350.s002.docx]

**Responses to reviewer’s comment on**

**PLOS Computational Biology (PCOMPBIOL-D-21-00540) - [EMID:e658e7d7250765d2]**

**Response to Editor’s Comments**:

1) One of the most pressing issues raised by reviewers is the details about the data and software, data and code availability to reproduce the results, and code access to be able to apply the proposed methods to other datasets.
***New details in the paper***

2) I hope the other comments from the reviewers are also useful to make your work more accessible and relevant to others.

***I really appreciate the time and detailed effort put in by the reviewers.***

***Actually, this has been one of the most enjoyable papers on which I have undertaken a major revision. The comments from all reviewers has made us really re-examine what we have said and done.***

Comments to the Authors: Please note here if the review is uploaded as an attachment. **–**

***Review is uploaded as an attachment***

**Responses to Reviewer's Comments**

***I had the standard (continual crashes) when trying to use Word’s review mode to keep track of the large number of changes in response to the reviewer’s comments and suggestions. I instead outlined in grey areas where changes were made.***

Reviewer #1: The Authors present a comparison of several DTWA algorithms, along with a method for ensemble voting to better match a consensus signal with a "gold standard" signal generated from the sequence of synthetic controls. While the ensemble voting and comparison methods themselves appear sound, and the next steps in using this method to detect modified bases are indeed exciting, and I have confidence will probably work quite well, I found it difficult to review and validate.

My most pressing issues with the current submission, is the lack of raw data and which software (and versions) were used to generate the "gold standard" squiggles, and read event tables. I checked the cited publications, and they also did not mention this information (which should have been picked up before now!). Without this information and data, the methods presented are not reproducible, and therefore do no meet the publication requirements. It also makes it difficult for me to review some of the finer points of the methods. The segmentation algorithms and models used in basecallers have changed many times over the years. These have a direct impact on the results of the methods presented, and their further use by the Author's peers. Furthermore, the code provided as part of the package for "data" is from a different publication, and no code for the methods presented have been provided. This is simply unacceptable for a computationally based manuscript. At the very least these issues should be resolved.

***This detail has been added to the paper – and code and data uploaded. Rewriting and testing the code has provided the opportunity to clarify documentation – although the tool is still a research version unoptimized for speed***

I go into more detail on these points as well as others in my review below to help speed up peer review in the future, but as it stands, the work is not reproducible.

I do hope the authors are not discouraged by my review, as I do think the direction of the work is interesting, exciting, and publishable with work on the areas mentioned.

***<SMILEY> Thank you for your concern. I am not discouraged. I always tell my students – “If I can make that number of comments on your paper, then it is a clear indication that, overall, I understand what you are trying to say and think it worth saying!”</SMILEY>. Actually, this has been one of the most enjoyable papers on which I have undertaken a major revision. The comments from all reviewers has made us really re-examine what we have said and done, and has flipped the paper on its side in a number of places.***


The title:
"generate improved consensus signals from nucleotide sequences produced by a nanopore device"
The consensus signals are not being produced from nucleotide sequences, they are being produced from the raw signal segmented into events from nanopore sequencing reads.

***Similar comment by Reviewer 2
Agreed – they are not produced directly by the device. However, they are produced by an algorithm that uses the signals produced by the device. Have changed the title to better reflect multi-stage operation***

***Evaluating the effectiveness of ensemble voting in improving the accuracy of consensus signals produced by various DTWA algorithms from step-current signals generated during nanopore sequencing

Short title: Improved step-current signal consensus by combining DTWA with ensemble voting***

***Have modified the abstract to fix same concern***

Fig 1. Image is quite low in resolution (as are all the figures)
Leader sequence difference to the rest of the RNA signal is not easily resolved with the lack of height in each signal. It may help the reader understand how the leader/DNA adapter was detected and trimmed from the description if they can see the striking difference in signal on either side of the PolyA tail.

***Followed reviewer’s suggestion, I have made difference between leader and rest of data clearer in a new picture – much improved.***

***Unclear of why the resolution problem. One issue might be that the high information content may make the picture look fuzzy. I have decreased the number of lines displayed in an attempt to solve that – but that fixes vertical resolution issues and not horizontal***

***I have regenerated pictures and validated with PACE as required.***

75: "The state of the practice is to use black box neural networks (5) or hidden Markov models (6) to turn the
raw current signal into nucleotide sequences."

This isn't entirely correct. There are many examples, including from ONT, of event segmentation methods.
for example, the segmentation code from <https://github.com/nanoporetech/scrappie> is freely available.
Also, the latest basecallers (bonito for example), don't use event tables as as such, working from the raw signal using 1D.
There are also other methods of signal segmentation, such as that used by cwDTW, or any number of open source basecallers (Nanocall, DeepNano...)

***Addressed in the text – lines 59 - 63***

2.1 Data generation

Please note the MinKNOW version as well as the "OEM provided current to signal squiggle converter" name and version.
Presumably you are referring to Guppy, although I can't tell how old this RNA001 data is, so it could even be Albacore.
Either way, the metadata for version information can be found in the metadata of the .fast5 files.
The reason this is important, is because the algorithms for signal segmentation have changed a number of times, and without knowing the particulars, your methods are not reproducible.

***Addressed in the text – Lines 134 - 156***

On that note, the only data provided seems to be flat .txt files with the (i think) event values, with no readID or other identifying features. fast5 files containing the raw signal data to work with in order to generate these event tables, should be included. Again, this is to ensure the methods are reproducible.

***Information and code will be uploaded to Github/nodroglual to convert files of other formats into the text format used in this research tool.***

***The conversion code was in the process of being uploaded as of 27 July 2021. Some of the original Fast5 files are on a remote server which not allowing itself to be accessed. These files will be uploaded as soon as the server problem is solved.***

***Could the reviewer please clarify whether all fast5 file information should be uploaded or just enough to demonstrate that the conversion code correctly converts such information into the simpler text file format used by the GetGoldStandardInfo_Plos1_CompBio_July2021( ) and GetStreamFromFile_Plos1_CompBio_July2021( )***

Further to this, the original OEM kmer models are not included, and also cannot be derived, for as previously mentioned, no software or version information is given. Only a "gold standard" file is produced. This makes the gold standard generation not reproducible as well.

The base sequences of the particular Sequin and Enolase spike-in controls used should be provided as a supplementary, as this is what is used to produce the "gold standard" from the kmer models.

***Addressed in the text – Lines 134 – 156 - uploaded to Github.com/nodrogluap***

138-143: The files containing the code which does this such as "RemoveHeader_10Jan2019.m" should be referred to. This applies for all sections that refer to computational methods. For reproducibility, the user should be able to retrace the Authors steps.

***Code has been refactored and tested, and then uploaded to Github.com/nodrogluap/DTWA. I started referring to thisFile.m and thatFile.m in the text with , but did not find it useful.***

***To solve this problem –***

1) ***Have added Fig 2 – a schematic of the process in Section 2 - Methods***
2) ***Add details of the main stages of the process – Lines 438 – 447***
3) ***Provided scripts in nodrogluap/DTWA to show how all the figures can be generated***
4) ***Provided a basic document in nodrogluap/DTWA to explain what part of the code does what***
5) ***All code has been retested and documented but remains un-optimized for speed***

168: Wouldn't mapping the nucleotide sequences allow for the detection of chimeric reads? multi-mappers or reads with multiple primary alignments with high mapping score would indicate this. This ideally would be done first, to filter the read IDs which give full length sequences, of which to then remove headers, and potentially malformed tail sequences (not currently assessed in this analysis). Then the resultant squiggles used for DTWA, as they have the highest chance of matching the gold standard.

Doing everything in squiggle space only seems to miss the advantage of having sequence information available, especially if the goal is to do modification detection in the future. The only reason to need to do everything in signal space would be if it was being used for readUntil/adaptive sequencing, which based on the methods, was not considered (using the tail end for setting mean for leader trimming, using matlab)

***In the paper we discussed what might be causing some of the distortions remaining in the cleaned Squiggles. Whether these are, or are not, primarily caused by chimeric reads is part of our on-going research. However, the cause of these distortions is not germane to this paper since they are removed in the next stage of our proposed cleaning process.***

2.2 DTWA Algorithms and proposed success measures

195: The OEM provided nucleotide-to-picoamperage mapping should be named, and provided, and referred to here.

***Have inserted as requested***

249-254: if there is a limitation of "inserting events", why not go back to the raw signal where the events were generated from? If a particular event was generated from a rather large section of raw signal, the number of inserted events could be estimated, or an orthogonal segmentation method from any number of open source tools could be used to attempt to insert the correct events.

***We have added a new Section 7 to discuss this issue in detail. As described in the text – the problem has never been ‘how to decide’ something is missing, but to decide what is a valid thing to insert without generating false positives when we look for chemical modifications in our next stage of research into applying statistical analysis to squiggle ensembles.***

Results:

Figure 2. The blue box showing similarity of signals, is not entirely clear given the scale of the figure. A better representation fo this would be to zoom that to a full supplementary figure, colour each squiggle differently, and overlap the plots. This can get messy, so perhaps only half of the selected squiggles need to be used. As it stands, I can't tell any similarity.

***Comment was meant as a general aside that if the reader “looked carefully” there were hints of similarity already appearing***

***Agree that the comment was not helpful given the resolution (and the need to be familiar with what was “supposed to be there”). Blue box and comment deleted.***

***New figures generated. Changing the number of streams displayed fixes vertical resolution but the high information content still make horizontal resolution look fuzzy.***

3.2.1 Enolase squiggle investigation

331-332: Is this code provided? if so, which files. If not, please include, along with how the timing was measured for all methods. There is no way to reproduce the timings without this.

***Have inserted as requested***

3.2.3 Qualitative analysis of the DTWA consensus characteristics.

378:380. The x1.7 longer squiggles, as stated by the authors previously, has not been checked against other datasets. It is also contingent on the older RNA001 dRNA kit (RNA002 is the current kit), and the various cleaning metrics rely on the segmentation method used, which is currently unknown due to the lack of information or raw data from the authors.

The gold standard is also unknown, as the model used to produce it is not mentioned or provided. How this model is then converted into the gold standard text files with picoamp event data points, is not provided, and thus unclear.

***This reviewer’s comment made us realize that in a number of places we were saying one thing and then successfully doing ‘the-something-else’ that we actually wanted to do. We therefore did some significant rewording in the revised document***

***As Figure 2 shows, the gold standard plays no role in header removal, consensus generation or voting.***

***The fact is – consensus generation is NOT an attempt to generate a gold standard approximate from the ensemble, but to generate an average of the ensemble from the majority of its members to better find the chemical modifications present in the minority of its members. The only role the gold standard plays in any of the processes is in the metrics as the ‘best estimate’ of the unknown average of the ensemble that can be compared to the consensus estimate of the ensemble average.***

Figure 4 (and most of the comparisons between time warped squiggles): When comparing time warped squiggles. Stacking squiggles is a rather clunky way of demonstrating the similarities/differences between them. Overlapping signals is one way (though still quite messy). Plotting the dtw paths, especially plotting each method's consensus vs gold paths, would not only give a measure of distance, but also if the methods are consistent across the various warped regions of the reads.

It should be much clearer to assess the various approaches and demonstrate the various features of each method. (are certain regions handled better/worse for each method?/Is there limitations to how much warping can be corrected?/is there a better method other than global scoring?/etc).

***The basic reason why we did not put warp path pictures into the original paper was the following.***

***There is gold standard information underlying each distorted squiggle and the distorted consensus not found in other research fields using DTWA ideas. We felt that this fact meant that if we plotted warp paths then we would get almost a straight line since one signal (the gold) would be essentially equivalent x = 1 : 2000 and the other an almost equivalent signal 1.7 times longer on average y = 1 : 3400 + noise.

However, since the reviewer queried the absence of the warp path information, we considered it likely that other readers would query the same thing. Therefore, we introduced a new section on consensus analysis tools for this new squiggle environment***

- ***three different type of warp path displays – see Figs 5, 9 and 13***
- ***A modification of the Matlab dtw( ) figure to characterize the difference between consensuses and between them and the gold standard – see Figs 4 and 8***
- ***In addition, we have explained why our original ‘stacked’ method is a useful complement to the other tools. The main feature of stacking is that we can see, in addition to warp path information, residual amplitude differences between consensus signals visually Figs 6, 10 and 14 – and in a very precise manner the common differences of each DTWA consensus to each study’s individual gold standard in the new Fig. 15. An issue we believe introduces a whole new issue that could have general implications in squiggle analysis.***

You may also consider using something other than matlab to plot this data, as the quality of matlab plots is quite poor. Python or R have libraries with a reputation for producing clearer plots for publication, and are also free.

***That was the fault of me taking the ‘initial free format’ of PLOS Computational Biology as applying to picture format as well as text and using .png format required in other journals rather than verifying .tif files through PACE.***

5. Conclusion

562: Identification of a potential chemical nucleotide modification would be relatively straight forward to demonstrate using the current method, by analysing any of the number of dRNA modification datasets now published, with modified and unmodified controls. producing the consensus against the gold standard for untreated, then compare the treated reads against it. By comparing the warp paths and mapping that back to the associated base, a measure of modification can be deduced. This is indeed an exciting path forward.

***This is the direction we are planning to explore in our next research stages. However, some of your comments have challenged us to have a closer look at some of our results, leading to insights of new issues– see Section 7, Fig. 15. We will need to solve the problems about unexpected COMMON differences between DWTA consensus signals and the gold in these simple examples before moving on to more detailed verification.***

Spelling/grammar
100: identity -> identify **-- *Corrected***
131: enblign -> enabling (i think?) – ***Sorry -- confusing typo – end-align***
182: averaged -> average **– *averaged was the word wanted – rephrased sentence to avoid confusion***
538: nanosequencer -> nanopore sequencer - ***Corrected***
538: nanostream - > datastream/signal **-*– changed nanostream to nanopore-stream – 4 items***

**Reviewer #2**:

Overall this is a well-written paper

**– *THANK YOU, I hope you will make the same comments with all the changes* 😊 –**

that I think will benefit from making the technical methods clearer. I think other reviewers may be more qualified to comment on the methodology of the data generation and new evaluation metrics. But I think this paper reads well and, to my knowledge, represents a novel contribution to the field.

- If I understand the paper correctly, voting is used to improve a single DTWA algorithm; in particular, it is NOT used to combine the results of several DTWA algorithms. Is this correct? If so, I think this should be made crystal clear; it took me a couple of readings to understand this.

***Similar comment by Reviewer 1 –***

***Changed title -- Evaluating the effectiveness of ensemble voting in improving the accuracy of consensus signals produced by various DTWA algorithms from step-current signals generated during nanopore sequencing

Short title: Improved step-current signal consensus by combining DTWA with ensemble voting***

***The new Fig 2 makes it more obvious that this is a comparison between DTWA methods rather than a combination, although an investigation of combination is under investigation***

- Line 187 - line number is in the middle of the equation This is a problem for all of the equations

***That appears to be a word to pdf conversion problem associated with Mathtype entries as it is not an issue with the word document. Will try to fix with the pdf drafts – but should not be a problem when published as line numbers are removed.***

- Section 2.2: I think this paper would benefit greatly from giving more background on how a consensus is generated. I assume these three algorithms take the noisy squiggles as input, compute a consensus squiggle that optimizes a particular objective function. I think this should be explained; and in doing so, foreshadow what is missing from these
algorithms that voting can fix.

***Agreed – summary of concepts behind how the DTWA algorithms function and expected differences has been added Section 2.2 DTWA algorithm characteristics and related comments now occur throughout the text***

- Line 186: it says that the goal is to minimize the mean and standard deviation of the DTW distance. Immediately following is equation (1). This wording is somewhat confusiong; equation (1) shows how to compute a single number, so what would be the mean and standard deviation of this sone number? My guess is that equation (1) is the formula for the mean that is being minimized; if this is correct, consider adding another equation with the formula of the standard deviation being minimized. If my interpretation is incorrect, then this paragraph needs more explanation of what you are taking the mean/standard deviation of when measuring the success of an algorithm.

***New equations added and surrounding explanation refactored***

- 198: "3 x 3 = 9 datasets" To be clear: this means that for each of the 3 algorithms, and each of the 3 datasets, you applied the algorithm to that dataset, to generate 9 different consensus sequences, correct?

***Correct – section reworded to avoid confusion***

- Line 201: It is unclear to me what you mean when you say "comparing DTWdist_{gold -> ensemble} against DTWdist_{consensus->ensemble}". In a "good" consensus, should the two metrics be similar? Should the consensus be much closer to gold than to the ensemble?

- Line 206: What is DTWdist with no subscript? I was onder the impression that the DTWdist metric should have a subscript delineating what two sequences (and/or sequence ensembles) are being compared. Perhaps DTWdist = DTWdist_{gold -> ensemble} - DTWdist_{consensus->ensemble}? If so make this explicit.

***The lack of a subscript was meant to imply that the concept was applied generically across all measures. Have modified wording to avoid confusion***

***We normalized each of these metrics,*** $nMetric=Metric/gold\_length$***, by the known gold-standard squiggle length for a given ensemble to provide a better tool for comparing several DTWA algorithms across multiple DNA and RNA studies.***

- Lines 238-245: Please explicitly define the measures you used to evaluate the results of voting

***The metrics were explicitly explained, but the wording did not make that obvious – have rephrased***

- Line 339: font size for "nDTWdist_{gold -> ensemble}" is off **- *FIXED***
- Line 349: font size for "nDTWdist_{consensus -> ensemble}" is off **– *FIXED***

- Line 461: This is the standard deviation across 7 different consensus signals (before after voting) - correct? If so please make this explicit.

***This was not what we intended to imply. Only the Enolase data set was larger enough (7000) to support the generation of multiple consensus signals. This section has been modified to reflect it was designed as a general overview of the similarity between the characteristics of consensus of small ensemble sizes (around 256) and the previously confirmed characteristics of a larger data set (7000) capable of supporting the generation of multi-consensus.***

- Results and (especially) conclusion are well-written – **THANK YOU**

**Reviewer #3:** The authors propose an optimization application supported by theory, with illustrative validations.

Comments:
1. Please edit the paper carefully such that to respect the instructions for authors. A homogeneous style is desired.

***We have adjusted the size of abstract, reformatted figures into .tif and checked with PACE, showed the difference between figure and table titles and captions, checked font sizes for headers.***

***If we have still missed something, please let us know.***

2. You should present the contributions with respect to your past papers that should be cited.

***I hope that the major rewrite and re-organization of the paper has addressed this concern***

Your past algorithms are very well appreciated. **– *Thank you***

3. The optimization problem is not defined. You are speaking several times about optimization and also including optimization algorithms but an overall optimization problem is not given

***We have rephrased the text to make the following clearer to the reader.***

***(1) We investigated three DTWA algorithms which used their author’s optimization approach to generate an average of an ensemble of time-distorted signals with many imperfections.***

***(2) We used voting agreement between noisy members of the ensemble to remove the imperfections with the expectation that that the optimized consensus would have the minimum dtw* *distance to the ensemble as a whole.***

4. As mentioned in the comment 3, the definitions of the optimization problems must be treated with attention. The authors are advised to include the following representative applications of optimization problems and algorithms as they are successful in various fields: Gene finding in the chicken genome (BMC Bioinform 2005), Second order intelligent proportional-integral fuzzy control of twin rotor aerodynamic systems (PCS 2018), Whale optimization algorithm for performance improvement of silicon-on-insulator FinFETs (IJAI 2020).

***In reference to our reply to query #3***

1) ***The success of the DTWA algorithms in other research fields is well established.***
2) ***We show that the approaches provide poor results in this new high-entropy squiggle environment and propose a solution.***
3) ***Intuitively we would expect the distance minimization with respect to the ensemble would occur when the consensus length was nearly the same length of the known input RNA molecule / gold standard model. We did find this as per Figs 11 and 12***

5. The connection between the optimization algorithms and the optimization problem is also not pointed out.

***Extending our response to 3***

***We have added a section outlining the basics of optimization technique behind the DTWA algorithms. The paper discusses the use of existing DTWA algorithms to optimize the production of an average of time distorted signals when standard averaging techniques fail. The success of these algorithms in other research fields is well established.***

***However, we show that the approaches provide poor results in this new high-entropy squiggle environment. We demonstrate that our new voting procedure is consistent in greatly improving the results from these existing three DTWA methods on three different samples physically prepared by different methods.***

6. The connection between the application section and the previous theory is not clear enough. More details are necessary for improved transparency.

***I hope that the major rewrite and re-organization of the paper has addressed this concern***

7. You should save the code of programs and examples, and cite the link to them in the paper. This is useful for validation, and helps the above comment. The importance of this comment is related to the fact that similar optimization algorithms are reported in the literature, they report excellent results but cannot be tested.

***The link to data and code has been updated. The code itself has been refactored and tested with added demonstrations on how to generate each of the figures in the text***

8. You should specify which are the parameters of the optimization algorithms, which of them should be selected by the user and which of them are random.

***The second reviewer made a similar comment – Have added section on details on the DTWA algorithms and things that impact the consensus.***

***We have shown that the DTWA algorithms, as coded by the original authors, do very poorly in this new squiggle environment because of the unique characteristics of the high entropy signals not found in other research fields using DTW and DTWA ideas. However, adding voting based on across ensemble agreement makes the results very consistent.***

***In fact, overall consistency and the new Fig. 15 leaves the reader (and us) with a related question –Why are the results so consistent between the DTWA consensuses developed by three very different coding approaches that they have common differences along their entire warp path with the gold standard that (supposedly) underlies each distorted squiggle?***

9. I am not sure if the comparison is correct because all algorithms used in the comparison including yours depend on parameters. Other parameters lead to other results.

***I am not sure what is meant by ‘depend on parameters.***

***One parameter that can impact the algorithm is the manner in which the optimized warp paths are calculated, and each of the DTWA* *algorithms use the same step pattern***

***The gold standard model specific to each ensemble study is not a parameter as neither the DTWA consensus generation nor the voting make use of the gold standard.***

***The cleaning of the squiggles was made as loose as possible – things had to be at least 3 standard deviations from a mean before being rejected -- meaning 99% of the data is retained.***

***The only ‘other parameters’ than could lead to other results is the use of random selection in the SSG algorithm and the impact on the order of squiggles on the SSG and MM algorithm and the use of different squiggle groups as is now discussed in the paper.***

***In Fig 11, the 20 consensus signals that initially had different characteristics directly from the DTWA algorithm have very similar characteristics after voting***

10. The stochastic effects are not reflected in the results.

***We have added a number of new visualization tools to better demonstrate the success of our method. Could the reviewer provide more information if something more detailed is needed***

Concluding, the paper has a strong potential for being appreciated and cited, but it requires improvements and also extension.

***We have been provided by the three reviewers with an excellent path to improve the paper. We hope our interpretation of how to solve their concerns meets with approval***

**Have the authors made all data and (if applicable) computational code underlying the findings in their manuscript fully available?**

Reviewer #1: No: The fast5 files, containing the raw signal data which the event values (mean value from signal segmentation) are not available. The code provided is from a different publication (Evaluation of Simulation Models to Mimic the Distortions introduced into Squiggles by Nanosequencers and Segmentation Algorithms) and is written in matlab (software requiring a rather expensive paid license). The code for this paper is not provided, and not maintained in an open repository, such as github/gitlab/etc.

Reviewer #2: Yes

Reviewer #3: No:

***Code has been refactored and data uploaded to github as suggested.***

***The previous code subroutines generated simulated data and then analysed the simulated data.***

***We use the same tools in this paper to analyse the effectiveness of th* DTWA *algorithms. Scripts have been added to show how the same subroutines can be called to analyse ‘real’ data. And generate the paper’s figures.***
